# Effect of cigarette smoke on gustatory sensitivity, evaluation of the deficit and of the recovery time-course after smoking cessation

**DOI:** 10.1186/s12971-017-0120-4

**Published:** 2017-02-28

**Authors:** Fabrice Chéruel, Marta Jarlier, Hélène Sancho-Garnier

**Affiliations:** 1 Fondation JDB Prévention Cancer, Espace Prévention Santé Antéïa, 2/4 rue du Mont Louvet, 91640 Fontenay Lès Briis, France; 2 Université Paris Sud, Université Paris-Saclay, Orsay Cedex, 91405 France; 3 Biometrics Unit, ICM - Montpellier Cancer Institute, Montpellier, France

**Keywords:** Tobacco smoking, Taste disturbance, Taste recovery, Electrogustometry

## Abstract

**Background:**

Study results have shown that chronic exposure to cigarette smoke affects the taste function in humans. However, neither the quantitative impact on taste sensitivity nor the time-course of taste recovery on stopping smoking have been precisely examined.

**Methods:**

The experimental design included 2 phases, (i) a case–control phase comparing the taste sensitivity level measured by Electrogustometric (EGM) thresholds from various parts of the tongue (locus) between smokers (*n* = 83) and non-smokers (*n* = 48), (ii) a follow-up study looking at the taste sensitivity recovery in smokers after smoking cessation (*n* = 24) and compared with non-smokers.

**Results:**

Smokers exhibited significantly lower taste sensitivity than non-smokers - the higher the nicotine dependence (Fagerström scores), the lower the taste sensitivity. After smoking cessation, EGM thresholds decreased progressively, and reached the taste sensitivity range of non-smokers depending on locus and time. After 2 weeks a recovery could be observed on the 3 Tip and the 2 edge loci; the recovery in the posterior loci was complete after 9 weeks, and in the dorsal loci recovery was observed only after 2 months or more.

**Conclusions:**

Smoking cessation does lead to a rapid recovery of taste sensitivity among smokers, with recovery time found to differ based on the sensitivity of loci of the tongue. The use of EGM could potentially be explored as a motivational tool for smoking cessation.

## Background

Smoke aerosols produced by burning tobacco contain a complex mixture of thousands of constituents [1, 2] such as irritants, carcinogenic molecules, heavy metals, carbon monoxide (CO) and psychoactive alkaloids, including nicotine. Some of these components may affect taste sensory mechanisms either locally on receptor, transduction, sensory cells, neuronal levels or more centrally, all resulting in a sensory deficit.

Significant taste sensitivity changes have been previously observed in smokers, using electrogustometry [3, 4]. Associated contact endoscopy has confirmed morphological differences in taste buds and vascularisation in fungiform papillae [5]. Determination with tasting solutions has showed that smokers’ detection thresholds were significantly elevated for salt, acid, sucrose or quinine [6–9]; but some other studies showed discrepancies or inconclusive results [10, 11]. The chronic use of smokeless tobacco appears not to substantially affect the gustatory function [12];

Other factors affect taste sensitivity, such as medication [13–15], inner ear surgery [16] or dental deafferentation [17]. These factors were not usually considered in previous studies and may be the cause of discrepancies between studies. The affected regions of the tongue have never been specifically tested. Moreover, the constant and short-time taste cell renewal, discovered by Beidler and Smallman [18] and recently documented [19–22], lead us to expect that the negative impact on taste would reverse after tobacco cessation. The eventual taste sensitivity recovery after tobacco cessation has not been explored, nor has the time-course of taste recovery after smoking cessation.

The difference in taste induced by tobacco consumption has serious implications on the nutritional status of smokers, as it results in poor eating habits [23]. Generally, the dietary habits of smokers are characterized by higher intakes of energy (cholesterol, saturated fat and alcohol), and by lower intakes of antioxidant vitamins and fiber (fruits and vegetables) [24, 25].

Tobacco users are generally unaware of the effects of tobacco on general health, oral health and oral cancer [26]. The effect on sensory perception and the demonstration of its deficit to the subject might reveal an actual threat the smoker may wish to avoid. Hence, taste evaluation may result in a tool reflecting a multi-factorial smoking toxicity both through permucosal and systemic access. Taste evaluation may also be a tool which exhibits a rapid deficit in order to impress the subject with an objectively measured effect of smoking on his/her own body. If we can measure this deficit and an eventual reversibility as a result of weaning, the subject might be rewarded by an objective recovery after smoking cessation. Consequently, measuring the deficit and the recovery of taste after weaning might help smokers to stop and to not start again.

Our study’s objectives are to investigate the smokers’ taste deficit as compared with non-smokers and to observe whether there is a reversible taste deficit related to stopping smoking.

## Methods

### Design

The experiment design included 2 phases:

- Phase 1: initial case–control phase comparing the taste sensitivity level of various parts of the tongue between smokers and non-smokers,
- Phase 2: A follow-up study during 6 to 12 months, looking at the variation of taste sensitivity in smokers after smoking cessation. Taste levels in previous smokers were compared to the basic non-smokers’ levels throughout the follow-up.

### Population

The subjects (smokers and controls) were recruited by advertisements on the campus of the University Paris-Sud and the INRA Research Center. All the attendees also met the investigator within the Consultation of Tobaccology of University Paris-Sud. The study was approved by the local medical authority (Preventive Medicine).

Included subjects (ages 18–64 years, 85 female and 46 male, 83 smokers and 48 non smokers) exhibited no mucosal lesions, mycosis, burning mouth syndrome, aphthous stomatitis or tongue piercing. Subjects under medication were excluded (except for oral contraception) as well as when presenting numerous dental deafferentations (root canal treatment or tooth extraction). Poly-addicted patients (tobacco + illicit drugs, tobacco + alcohol) were detected using standard tests [27, 28] and also excluded.

During the first visit, a 45 minute presentation was given to smokers and non-smokers. During this visit, subjects received full information on the proposed research and follow-up procedures. After obtaining the subjects’ oral consent, the visit included also a measurement of EGM taste sensitivity in all subjects (smokers and non-smokers) and an evaluation of the carbon monoxide (CO) level in expired air in smokers. Further visits of smokers included a consultation with the tobacco cessation specialist and an evaluation of the CO level prior to the taste sensitivity test.

During the first visit all smokers were offered help with tobacco cessation; the candidates for stopping smoking were invited back every week for 2 months, and then every month for 12 months. Various ways of smoking cessation were proposed (nicotine replacement therapy and/or behavioral and cognitive therapies) depending upon the results of the Fagerström Nicotine Dependence Test (FNDT) and the CO level; a nicotine replacement therapy prescription was proposed. This prescription was given for 3 months after the day of cessation in doses adapted to the patient, usually decreasing from 21 mg to 7 mg over 3 months.

**Fig. 1:**
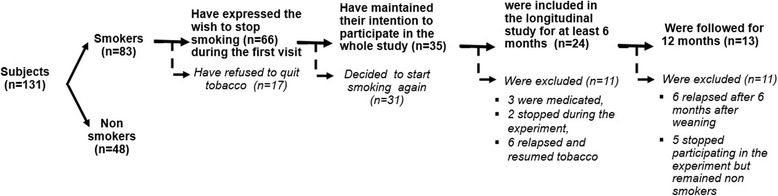
Flow sheet describing groups and drop out

In total, among the group of smokers, 66 subjects expressed their wish to stop smoking during the first visit, and only 35 achieved their objective and were included in the follow up study for at least 6 months. During the follow-up, 3 patients were medicated and excluded, 6 relapsed and 2 were quickly lost from the follow-up. In total, as shown in Fig. 1 (flow sheet), 24 smokers participated in the follow-up study for at least 6 months, with a weekly EGM test for two months and then every month for 12 months (*n* = 13) after the day they ceased smoking.

### Test sensitivity evaluation

Controls and smokers were asked not to drink anything (coffee, alcohol, soda, etc.) other than water, not to eat chewing gum or spicy food, and not to brush their teeth for at least two hours before threshold evaluation. Smokers were asked to stop smoking at least 2 h before threshold evaluation.

Electrogustometric (EGM) stimulation consists of iontophoretic stimulation. In our study, an anodal current [29] applies the cations (e.g.: Na+, H+) of the subject’s own saliva on the ionic taste receptors (ligand-gated trans-membrane ion channels) [30] involving ionotropic transduction mechanisms. The anodal current evokes sensations more often described as sour, salty or metallic [31] which suggest a gustatory function (taste threshold) [5, 32]. By contrast, a buzzing vibration, tingling or electrical sensation [32] suggest a trigeminal function [5]. As a result, the value for the lowest anodal stimulation at which the metallic or sour/salty taste was perceived was recorded as the taste threshold value [5, 33–36].

**Fig. 2:**
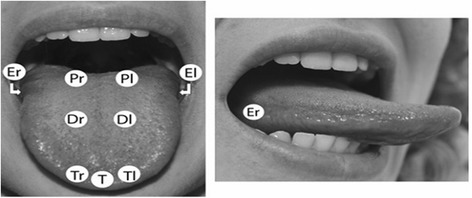
Location of the 9 recording loci on the surface of the tongue. T, tip; Tr and Tl, tip right and tip left (fungiform papillae); Er and El, edge right and edge left (foliate papillae); Dr and Dl, dorsal right and dorsal left (fungiform papillae); Pr and Pl, fungiform papillae just anterior to the circumvallate papillae

The EGM threshold was recorded in 9 different loci on the subjects’ tongues (Fig. 2).

A custom-made electrogustometer delivers from 0 to 100 micro-Amperes (μA), through a constant current generator via a ten millimeter-diameter stainless steel spherical electrode on a 40 square millimeter area. For each threshold evaluation, the current was applied for 1 s. [5, 37].

The nine tongue loci were defined as follows:

- **Tip** of the tongue middle (T), right (Tr) and left (Tl), where the density of fungiform papillae is highest;
- **Dorsal** right and left (Dr and Dl), where the density of fungiform papillae is lowest;
- **Edge** right and left (Er and El) on the foliate papillae;
- **Posterior** right and left (Pr and Pl) just anterior to the circumvallate papillae; during a recording session, all loci were tested following the same sequence: T, Tr, Tl, then Er, El, Dr, Dl and Pr, Pl.

### Threshold evaluation

The electrode was put on the tongue so that it could adapt to the tactile-cool stimulation, then a current of intensity above usual thresholds was used to identify the sensation the subject should expect, (well below any somatosensory sensation), after which a null stimulation was done to better show the difference between null and positive. The EGM threshold was fixed as starting at 7 μA at the Tip, 12 μA on the Edge and the Posterior parts, and 20 μA on the dorsal parts of the tongue. The subject had to answer the question “did you perceive a sensation” (Yes/No)? Different intensities of current with a constant variation (1 or 2 μA steps on the Tip, 5 μA on the Edge or 10 μA on the dorsal locus) were applied successively following a staircase protocol until the subject could not perceive any sensation and finally a null stimulation was applied. The threshold evaluation was repeated starting from the preceding result but with a smaller intensity difference to precisely identify the EGM threshold value. The EGM detection threshold was taken as the lowest current intensity eliciting a metallic-like, sour or salty taste perception.

“Blind test” trials without current application were intermingled at random during the test session to ensure quality control of the answers. The test duration for EGM thresholds evaluation at all loci was about 15 min.

After each use, the EGM electrode was cleaned and sterilized (120°, 2 h).

### Data collection

At the second consultation each smoker was given a self-assessment questionnaire [38], validated by the French Society of Tobaccology. Collected data included gender, age, weight, height, and the smoker’s profile (history of smoking, age of starting smoking, number of cigarettes/day, and previous attempts to stop smoking for at least seven days). This questionnaire included an evaluation of the motivation to cease tobacco, measured on a linear visual analogical scale from 0 to 10, an evaluation of the anxiety and depression levels using the Hospital Anxiety and Depression Scales (HAD test) [39].

The FNDT [40] indicating the dependence on smoking and a measure of the carbon monoxide (CO) level in expired air were recorded during sessions before tobacco cessation.

Reasons for stopping smoking as well as associated fears (fear of not succeeding, weight gain, mood changes or loss of intellectual or sensory faculties…) were also recorded. A short questionnaire for non-smokers (gender, age, weight, height…) was completed.

### Statistical analysis

Descriptive methods - qualitative (%) and quantitative (medians) - were used to present the various population indicators. Non-parametric statistics were employed because the threshold values did not follow a normal distribution. The comparisons between the sensitivity thresholds of the various groups were analyzed using the Kruskal-Wallis test (KW) and a linear mixed model covering each locus for a global comparison of repeated measures (The assumption of normality of residuals was not met, nevertheless a mixed model is still robust in terms of parameter estimation) and the Mann–Whitney U-test (MW) for individual loci comparisons. These analyses were done using the STATA 13 software (Stata Corp LP, College Station, TX, USA).

## Results

### Evaluation of smoking impact on taste sensitivity: difference between smokers and non-smokers

Table 1 gives the groups’ main characteristics. All smokers smoked light (flue-cured) filtered tobacco.

**Table 1:** Main characteristics of the groups

Characteristics	Total smokers (*n* = 83)	Non-smokers (*n* = 48)	Quit-smokers (*n* = 24)
% male	43%	25%	54%
Mean age male^a^ Mean age female^a^	35 [±13] 38 [±12]	34 [±17] 32 [±13]	42 [±8] 41 [±13]

^a^[±SD]

**Fig. 3:**
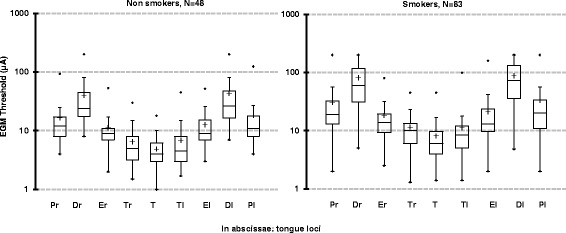
Effect of smoking on Electrogustometric thresholds. Box plot distribution of electrogustometric (EGM) thresholds (log) recorded in Non-Smokers (*N* = 48) and Smokers (*N* = 83) at nine loci on the tongue. (Each box plot presents from bottom to top: “•” = outliers, 5^th^ percentile value, first quartile (Q1), median, third quartile (Q3), 95^th^ percentile value), outliers; “+” (linear mixed model test *p* < 0.001)

The non-smokers’ thresholds were significantly lower than those of the smokers, either globally (*p* < 0.0002 with the KW test and in the linear mixed model *p* < 0.001 with a β = 0.52) or at each locus (*p* < 0.01 with the MW test); threshold dispersion was wider among smokers than among non-smokers (Fig. 3). Overall, values were increased by 90-100% on the right or left Tip in smokers compared to non-smokers, by 150-175% on the Dorsal loci (right or left) and by 50 –80% on the other loci.

### Influence on taste sensitivity of smoking characteristics

In the group of 83 smokers the median of cigarettes/day was 12 and the median smoking duration 17 years. The Fagerström score was distributed as follow:

- first group (0–2 Fag, *n* = 37) with a low dependence where the median of cigarettes/day was 6.5 and the median smoking duration was 9.5 years;
- second group (3–5 Fag, *n* = 24) with a median of 10.5 cigarettes/day and a median duration of 23.5 years;
- third group (>5 Fag, *n* = 22) with a median of 20 cigarettes/day and a median duration of 21 year.

Comparison of these 3 groups with the non-smokers group showed that the test sensitivity thresholds decreased as dependence increased: *p* (KW) = 0.0001 (Table 2). The decrease in the (Fag 0–2) group, compared to non-smokers, was not significant (*p* = 0.19), but the differences between the smokers’ groups as classified by the Fag test were all significant (MW test: *p* < 0.05).

**Table 2:** Taste sensitivity thresholds and Fagerström score

Group	*N*	Median (min – max)
Non-smokers	48	9.5 (4 – 31)
Fagerström 0-2	37	12.5 (2 – 160)
Fagerström 3-5	24	15.65 (7.5 – 56)
Fagerström >5	22	21.5 (4.8 – 92)

(*p* value (Kruskal-Wallis test) = 0.0001)

### Influence of cessation

#### Comparison between smokers’ and non-smokers’ thresholds after Quitting Day (QD)

**Fig. 4:**
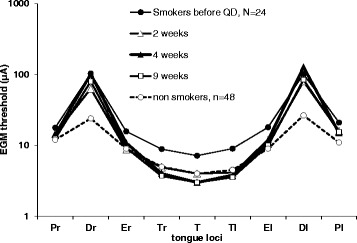
Variations of EGM thresholds during the first 2 months after quitting day. (KW test at 4 weeks: *p* > 0.41, at 9 weeks: *p* > 0.87)

After 2 weeks a “recovery” as compared to the non-smokers’ baseline could be observed at the 3 Tip and 2 edge loci (where the decreases were lower), the smokers’ thresholds reaching the initial level of the non-smokers and the global KW test on the 9 loci gives *p* = 0.41 (Table 3). The recovery at the posterior loci was observed after 4 weeks and was total after 9 weeks. Thresholds for dorsal loci (where the sensory loss was the most significant) were the only loci to remain high after 2 months but the global KW test on the 9 loci compared to the non-smokers’ baseline gives *p* = 0.88 at 9 weeks (Fig. 4). For the 13 smokers who were monitored for 1 year, it took 8 months for dorsal loci to reach the median value of non-smokers’ thresholds.

**Table 3:** Evolution of thresholds/loci, 2 weeks after tobacco cessation as compared to non-smoker

	Median (min – max)		
Tongue Loci	Smokers 2 weeks after cessation	Non-smokers	*p*-value
Pr	14.5 (5–141)	12 (4–93)	0.0802
Dr	63.15 (20–200)	24 (8–200)	0.0001
Er	8.7 (3 – 127)	9 (2 – 53)	0.6026
Tr	4.9 (1.7 – 30.5)	5 (1.5 – 30)	0.9727
T	4 (1.6 – 13.5)	4 (1 – 18)	0.6963
Tl	4.15 (1.4 – 31.5)	4.5 (1.7 – 45)	0.6185
El	10.5 (3 – 144)	9 (3 – 52)	0.5052
Dl	80 (23.5 – 200)	26.5 (7 – 200)	0.0001
Pl	15.75 (6 – 123)	11 (4 – 124)	0.0802

(*p* value by loci: Mann–Whitney U-test; global KW test: *p* = 0.41)

### Impact of gender on EGM thresholds

The effect of gender on taste thresholds was investigated for non-smokers and smokers. No significant difference was observed between men and women, both in non-smokers and in smokers (MW test: *P* value varied from 1 to 0.2 depending on the locus).

## Discussion

This study, using the electrogustometry method, showed the variability of sensory disturbances related to various loci on the tongue caused by tobacco smoke and the possibility of rapid rehabilitation of taste sensitivity after smoking cessation.

Compared to tests based on chemical solutions, EGM is a reliable tool for the clinical evaluation of taste sensitivity in clinical settings [13, 36, 41, 42]. The technique is especially appropriate for testing the whole taste sensory chain’s integrity including ionotropic transduction mechanisms, but excluding metabotropic transduction mechanisms because sweet bitter or umami taste transduction mechanisms rely on second messenger systems and are not activated by EGM stimulation (ionic stimuli) [43]. Thus electrogustometry cannot reflect the full taste experience accurately [44]. Nevertheless, the electrogustometry method is well adapted for a specific taste evaluation and gives precise and reproducible measurements of detection thresholds for a major part of taste sensitivity evaluation [16, 37, 45, 46].

In a recent work Pavlidis et al. [35] demonstrated a statistical difference in the EGM thresholds between the 2 genders in smokers and non-smokers. We have not observed such a difference between males and females in all tested loci. Along the same lines, Boucher et al. [17] with the same EGM device and the same method used in the current study did not observe any impact of gender on EGM thresholds. In our work, the tested loci are not exactly the same as those of Palvidis’ work, and furthermore, subjects presenting numerous dental deafferentations were excluded [17]. Thus these factors may be the cause of discrepancies between these studies.

In agreement with many other studies that have been published previously [3, 4] and more recently documented [5, 35, 47], significantly higher EGM taste thresholds were found in smokers compared to non-smokers, confirming the taste sensitivity deficit in smokers.

There are several hypotheses concerning the mechanism of taste sensitivity decrease: significant changes in shape, size and vascularisation of the fungiform papillae [5, 35], decreasing number of taste cells [47, 48], indirect result of tobacco substances impacting salivary glands [49–51], reduced zinc, vitamin B, E, and acid folic levels, all these components affecting taste [52–55]. One other explanation concerning the mechanism of taste sensitivity decrease is that nicotine acts at a central level and modulates the taste signal. Indeed, it was shown that the application of nicotine on the tongue surface modified the responses of the neurons in the nucleus of the solitary tract (NTS) of rats [56], the principal central relay in the gustatory pathway of taste buds of the tongue.

The mechanism of harmful effects of tobacco components on the taste system is not yet clear. Thus further studies are needed to identify in tobacco smoke the molecules responsible for the harmful effects on the gustatory function.

The dorsal loci were more impaired when compared to tip loci, which is consistent with the lower density of fungiform taste papillae at these locations [57, 58]; hence any deficit is more easily detected. Edge and Posterior loci seemed the least affected, and this might be a result of the relatively higher densities of these papillae and/or because of dual innervations: one by the glossopharyngeal and another by the chorda tympani nerves of the posterior loci [59, 60]. Such data suggests that areas behind the curve of the dorsal part of the tongue were protected.

Another main finding was to observe a statistically significant increase in EGM thresholds related to Fagerström score levels: the higher the dependence, the higher the thresholds. In the same way recently, Khan et al. [47], showed an inverse relationship between the fungiform papillae count and number of packs smoked per year (a quantification of cigarette smoking). The higher the number of cigarettes that were smoked, the lower the fungiform papillae count. As reported in a previous study, an inverse relationship was observed between the EGM thresholds and the number of fungiform papillae, in healthy subjects [61], in patients with a severe damage to the chorda tympani nerve or with chronic otitis media [33, 42], or in smokers [47].

Finally, our study demonstrates that the decrease in taste sensitivity in smokers is reversible when cessation smoking. The EGM thresholds decreased progressively, and reached the taste sensitivity range of non-smoking controls. The recovery was achieved 2 weeks after smoking cessation on the tip and lateral sites of the tongue. By contrast, at dorsal loci, it took 8 months to reach the same thresholds as non-smokers. In some subjects, individual thresholds at dorsal loci reached non-smokers’ levels only at or after 12 months.

Taste bud density could be responsible for the recovery timing but not the double innervations. Hence the areas with a high bud density recovered faster (anterior 2/3 of the tongue), than those with a low density (tongue’s dorsal part). The regeneration of gustatory sensory cells could initiate the functional recovery of taste, but if the time course of what was observed at the tip of the tongue is compatible with this hypothesis, it was not the case with the time-course of recovery observed in the dorsal sites.

Indeed, taste cells (epithelial origin) located in the taste buds are renewed continuously throughout life, with a 10-day turn-over on average [18, 62]. Futhermore, several studies [19, 22] using genetic lineage tracing methods on progenitor/stem cells for taste buds, suggested that cellular life spans within the taste buds may exhibit different longevities. Thus, Perea-Martinez et al. [22] suggested that different functional cell subsets may turn over at different rates, with half-lives of 8 to 24 days.

The exact mechanisms that regulate this continuous cell renewal are still not clear. Currently we have insufficient information to accurately explain how tobacco smoke disturbs the taste sensitivity mechanisms and how regeneration occurs.

In any case, this evaluation of taste impairment provides a rapid indicator of the harmful consequences of tobacco on smokers. Such findings could provide a motivational help to encourage smokers to quit tobacco and can be reinforced by the observation of taste sensitivity recovery. Concerning encouraging young people not to start smoking, this EGM tool could demonstrate to them in a concrete way the harmful effects of tobacco consumption. Delaying people’s initiation into regular smoking represents a major public health issue and could help to reduce people’s tobacco consumption. Therefore, further studies are required in this area to develop a randomized intervention trial to demonstrate the effect on stopping smoking and preventing the initiation to tobacco in young people using the electrogustometry tool.

## Conclusions

In this study we showed that the taste disturbance is frequent in smokers and varies with the tongue loci, that the intensity of such disturbance is linked to the intensity of smoking (number of cigarettes/day), and that smoking cessation leads to rapid recovery of taste sensitivity.

## Abbreviations

EGM: Electrogustometric
Fag: Fagerström
FNDT: Fagerström nicotine dependence test
KW: Kruskal-Wallis test
MW: Mann–Whitney U-test
QD: Quitting day
